# Exaggerated Increases in the Serum Cortisol Level in a Woman Following Oral Contraceptive Treatment

**DOI:** 10.1016/j.aace.2024.07.005

**Published:** 2024-07-14

**Authors:** Run Yu

**Affiliations:** Division of Endocrinology, University of California, Los Angeles David Geffen School of Medicine, Los Angeles, California

**Keywords:** hypercortisolemia, total cortisol, cortisol-binding globulin, oral contraceptive, estrogen

## Abstract

**Background/Objective:**

Extreme hypercortisolemia in an otherwise healthy patient can be due to familial dysalbuminemia, generalized glucocorticoid resistance, and estrogen-containing medications. I report a woman who appeared to have an exaggerated increase in the serum cortisol level following oral contraceptive treatment.

**Case Report:**

A 50-year-old woman presented with extreme morning hypercortisolemia—cortisol levels of 61 and 55 mcg/dL (4 and 3 months before presentation, respectively; normal range, 8-25 mcg/dL)—found during workup of mildly increased white cell counts. The morning cortisol level had been 10 mcg/dL after administration of 1-mg dexamethasone. The 24-hour urine free cortisol level had been normal and only slightly increased after correction by creatinine. The patient was anxious about the extremely high cortisol levels but otherwise felt well. She took norgestimate-ethinyl estradiol contraceptive (0.18/0.215/0.25 mg - 35 mcg). Physical examination showed a well-appearing, lean female. The thyroid-stimulating hormone, total thyroxine, free thyroxine, total triiodothyronine, free triiodothyronine, androstenedione, dehydroepiandrosterone sulfate, aldosterone, and renin levels were normal. Morning total cortisol and cortisol-binding globulin (CBG) were tested before and after she held the oral contraceptive for 2 months. The total cortisol and CBG levels decreased from 50 to 26 mcg/dL and from 6.4 to 3.8 mg/dL (normal range, 1.7-3.1 mg/dL), respectively.

**Discussion:**

Increases in the serum cortisol-binding proteins are a well-recognized cause for increases in the serum cortisol levels.

**Conclusion:**

This case suggests that modern oral contraceptives with low to moderate estrogen activity can cause extreme increases in the serum cortisol levels due to marked increases in the CBG levels.

**Highlights**

• Estrogen dose-dependently increases total cortisol level.
• Modern contraceptives usually only modestly increase total cortisol level.
• Contraceptives can cause extreme hypercortisolemia in a susceptible patient.
• Estrogen-containing medications are the most likely cause of hypercortisolemia.

**Clinical Relevance**

Modern oral contraceptives with low to moderate estrogen activity can cause extreme hypercortisolemia in a patient with endogenously increased cortisol-binding globulin and cortisol levels. In patients with hypercortisolemia of any degrees, medications containing estrogen such as oral contraceptives should be suspected as the most likely cause of hypercortisolemia.

## Introduction

The morning or random serum cortisol levels are often measured for various indications.^1^ The routine laboratory assay of cortisol measures the level of total serum cortisol including approximately 80% cortisol-binding globulin (CBG)–bound forms, 10% albumin-bound forms, and 10% free and unbound forms.^1^^,^^2^ Increased morning or random serum total cortisol levels (hypercortisolemia) are usually caused by stress, acute illness, pituitary or adrenal tumors, and medications and rarely by familial dysalbuminemia and generalized glucocorticoid resistance.^1^^,^^3^^,^^4^ It has been known for a long time that oral contraceptives are the most commonly used medications that cause hypercortisolemia through upregulating CBG levels.^5^^,^^6^ The effects of oral contraceptives on total cortisol and CBG depend on the estrogen activity of the oral contraceptives. Modern oral contraceptives with low to moderate estrogen activity only cause a moderate increase in the total cortisol and CBG levels.^5^, ^6^, ^7^ This case report describes a woman with extreme hypercortisolemia due to an oral contraceptive and endogenous mild increase in the CBG level. The patient’s case is relatively unusual because of what appeared to be an exaggerated CBG response to relatively low-estrogen-dose oral contraceptives.

## Case Report

A 50-year-old woman was referred to the endocrine clinic for hypercortisolemia. She had had mildly increased white cell counts (ie, 11 600/μL; normal range, 4000-10 000/μL) for approximately 5 years before presentation; during the workup of leukocytosis, the morning cortisol levels had been found to be extremely increased: (1) 61 mcg/dL 4 months before presentation and (2) 55 mcg/dL 3 months before presentation (normal range, 8-25 mcg/dL; Table). The morning cortisol level had been 10 mcg/dL after administration of 1-mg dexamethasone. The extremely high cortisol levels caused her anxiety. The patient denied centripetal obesity, facial plethora, weakness, proximal myopathy, psychological changes, easy bruisability, hirsutism, oligomenorrhea, or amenorrhea, acne, oily skin, abdominal striae, ankle edema, backache, vertebral collapse, fracture, polydipsia, or polyuria. She had had mild hypertension for many years. Her fasting glucose and hemoglobin A1c levels had been normal. The 24-hour urine free cortisol level had been normal and only slightly increased after correction by creatinine; the midnight saliva cortisol level had increased once; however, the saliva samples had been contaminated by blood on 3 other occasions due to gum disease (Table). She did not have a family history of pituitary or adrenal disease. Medications included diltiazem 120 mg daily and norgestimate-ethinyl estradiol contraceptive (0.18/0.215/0.25 mg - 35 mcg). Physical examination showed a well-appearing, lean female with normal vital signs except for the blood pressure of 141/80 mm Hg. Her body mass index was 19.7 kg/m^2^. She had a normal facial shape and complexion and proportional body habitus.

**Table:** Hormonal Test Results

Test	On OCP	1 mo off OCP	2 mo off OCP
Morning cortisol (8-25 mcg/dL)	61, 55, and 50	28	26
Morning ACTH (4-48 pg/mL)	25, 44	…	…
Morning cortisol after dexamethasone administration (<1.8 mcg/dL)	10	…	…
Midnight saliva cortisol (<0.112 mcg/dL)	0.222 once contaminated by blood 3 times	…	…
24-h UFC (<45.0 mcg/d)	35.8	…	…
24-h UFC (<32 mcg/g creatinine)	35	…	…
Cortisol-binding globulin (1.7-3.1 mg/dL)	6.4	…	3.8
Albumin (3.9-5.0 g/L)	4.4	…	…
Free cortisol (0.07-0.93 mcg/dL)	1.58	…	1.72
TSH	1.6-2.4	…	…
Total T4 (4.90-11.40 mcg/dL)	8.10	…	…
Free T4 (0.80-1.70 ng/dL)	1.00	…	…
Total T3 (85-185 ng/dL)	138	…	…
Free T3 (222-383 pg/dL)	296	…	…
Total testosterone (9-55 ng/dL)	13	…	…
Bioavailable testosterone (2.8-16.5 ng/dL)	1.1	…	…
Free testosterone (1.1 -5.8 pg/mL)	0.4	…	…
SHBG (17-125 nmol/L)	275	…	…
Androstenedione (0.26-2.14 ng/mL)	0.405	…	…
DHEA sulfate (400-3600 ng/mL)	582	…	…
Aldosterone (4.0-31.0 ng/dL)	7.2	…	…
Renin (0.5-4.0 ng/mL/h)	1.6	…	…

Abbreviations: ACTH = adrenocorticotropic hormone; DHEA = dehydroepiandrosterone; mo = month; OCP = oral contraceptive; SHBG = sex hormone–binding globulin; TSH = thyroid-stimulating hormone; T4 = thyroxine; T3 = triiodothyronine; UFC = urine free cortisol.

Cushing syndrome was deemed unlikely. Familial dysalbuminemia, generalized glucocorticoid resistance, and oral contraceptive use were considered to be potential causes of her extreme hypercortisolemia. Her thyroid-stimulating hormone, total thyroxine (T4), free T4, total triiodothyronine (T3), and free T3 levels were normal (Table), ruling out familial dysalbuminemia. The androstenedione, dehydroepiandrosterone sulfate, aldosterone, and renin levels were also normal, incompatible with glucocorticoid resistance. The bioavailable and free testosterone levels were low, and the sex hormone–binding globulin level increased, consistent with oral contraceptive use (Table). Total cortisol, free cortisol (measured by chromatography/mass spectrometry at Quest Diagnostics), and CBG (measured by radioimmunoassay at Labcorp) were tested before and after she held the oral contraceptive for 2 months (Table). The total cortisol level decreased from 50 to 28 mcg/dL 1 month after and 26 mcg/dL 2 months after she held the oral contraceptive. The free cortisol level increased (1.58 mcg/dL; normal range, 0.07-0.93 mcg/dL) while she was on the oral contraceptive but was even slightly higher (1.72 mcg/dL) 2 months after she held the oral contraceptive. The CBG level grossly increased (6.4 mg/dL; normal range, 1.7-3.1 mg/dL) while she was on the oral contraceptive but greatly decreased (3.8 mg/dL), although still higher than normal, 2 months after she held the oral contraceptive. She felt relieved after her cortisol levels considerably decreased after the discontinuation of the oral contraceptive. The patient was counseled that there is no known harm of high CBG levels and was given the options to restart the oral contraceptive or remain off it. Because she was already 50 years old, the patient opted not to restart the oral contraceptive.

## Discussion

Unexpected test results are frequently encountered in clinical practice.^8^ Some unexpected test results can be avoided at the first place if the appropriate alternative tests are ordered. In this healthy lean woman, the morning cortisol level was measured for unexplained mild leukocytosis; however, this is not the most appropriate test for hypercortisolism. In retrospect, 24-hour urine free cortisol testing could have been ordered as the screening test to assess whether hypercortisolism is the cause of mild leukocytosis. Because her 24-hour urine free cortisol level was normal or only slightly increased if corrected by creatinine, hypercortisolism could have been ruled out without testing morning cortisol. On the other hand, if the alternative appropriate screening test, low-dose dexamethasone suppression test, had been pursued first, her unsuppressed cortisol would have revealed her hypercortisolemia.

Hypercortisolemia in a healthy lean woman is most commonly caused by oral contraceptives.^1^^,^^5^, ^6^, ^7^ This patient’s extreme hypercortisolemia, however, was initially thought to be unlikely caused primarily by the oral contraceptive because hypercortisolemia is usually mild in modern times with the use of oral contraceptives with low to moderate estrogen activity.^1^^,^^7^ Familial dysalbuminemia is an autosomal dominant genetic disease caused by albumin mutations that increase the binding affinity of albumin to T4 or T3.^9^ Patients with familial dysalbuminemia are clinically euthyroid and have normal thyroid-stimulating hormone and free T4 or T3 levels but increased total T4 or T3 levels. Recently, patients with the R218P mutant albumin have been found to also exhibit hypercortisolemia because the same mutation also increases the binding affinity of albumin to cortisol.^3^^,^^10^ The degree of hypercortisolemia in familial dysalbuminemia can be as high as in this patient. Extensive thyroid function tests, however, revealed normal total T4 and total T3 levels and, thus, ruled out familial dysalbuminemia. Another cause of extreme hypercortisolemia without hypercortisolism is generalized glucocorticoid resistance.^4^^,^^11^ In this syndrome, inactivating mutations of the glucocorticoid receptor gene (*NR3C1*) results in stimulation of the hypothalamus-pituitary-adrenal axis. Patients with generalized glucocorticoid resistance exhibit normal or moderately increased adrenocorticotropic hormone levels, extreme hypercortisolemia, and extremely increased 24-hour urine free cortisol levels. The levels of mineralocorticoids and adrenal androgens are also increased by adrenocorticotropic hormone. In this patient, the 24-hour urine free cortisol level was normal or only slightly increased if corrected by creatinine, and the aldosterone and adrenal androgen levels were normal, effectively ruling out generalized glucocorticoid resistance.

After ruling out familial dysalbuminemia and generalized glucocorticoid resistance as the cause of this patient’s extreme hypercortisolemia, the oral contraceptive became the most likely suspect. Although estrogens are well known to increase the CBG levels in a dose-dependent manner, the exact mechanism of this phenomenon has not been clearly described but is probably through estrogen-stimulated transcription upregulation of the gene encoding CBG (*SERPINA6*).^1^^,^^5^, ^6^, ^7^^,^^12^ Oral contraceptives containing high-dose estrogen (equivalent to ethinyl estradiol ≥50 mcg) can increase the CBG and cortisol levels by more than threefold.^5^, ^6^, ^7^ Modern oral contraceptives usually have lower estrogen activity (equivalent to ethinyl estradiol ≤35 mcg) so that they increase the CBG and cortisol levels by 1.5-2-fold on average although with large and overlapping confidence intervals compared with those in women on progestin-only oral contraceptives.^7^^,^^13^, ^14^, ^15^ The estrogen effects on CBG and cortisol are completely reversible 6 weeks after oral contraceptive discontinuation.^16^ The decrease in the CBG and cortisol levels by approximately 50% after her holding the oral contraceptive for 2 months supports that this patient’s extreme hypercortisolemia is largely due to the oral contraceptive. Her slightly high CBG and cortisol levels after the contraceptive was held suggest that she had an endogenously increased CBG level for unclear reasons but possibly due to *SERPINA6* polymorphism.^17^

The patient’s increased free cortisol levels could not be readily explained by the effect of the oral contraceptive because patients on oral contraceptives with low to moderate estrogen activity usually have normal free cortisol levels,^5^ and her free cortisol level was even slightly higher after the oral contraceptive was held. Free cortisol assay is technically challenging so that the increased free cortisol level in this patient could be false positive.^18^ The values of the free cortisol index (total cortisol divided by CBG), a well-validated method of free cortisol estimate,^19^ were 21.6 and 18.9 nmol/mg before and after her holding the oral contraceptive, respectively, suggesting roughly similar free cortisol levels.

## Conclusions

This case demonstrates that modern oral contraceptives with low to moderate estrogen activity can cause extreme hypercortisolemia in a patient with endogenously increased CBG and cortisol levels. In patients with hypercortisolemia of any degrees, medications containing estrogen such as oral contraceptives should be suspected as the most likely cause of hypercortisolemia.

## Disclosure

The author has no conflicts of interest to disclose.
